# Acknowledgments

**DOI:** 10.4269/ajtmh.1124siv

**Published:** 2025-04-01

First and foremost, we thank the many study participants across the world who gave their time and effort to advance public health knowledge on COVID-19 and operational research. We also extend our sincere gratitude to the editorial team at the *American Journal of Tropical Medicine and Hygiene* – Editor-in-Chief Phil Rosenthal, Managing Editor Alison Jaeb, Editorial Production Manager Annie Calacci, and Peer-Review Coordinator Lori Young, for their invaluable support in publishing this supplement as well as the many manuscript reviewers who generously contributed their time and expertise.

Financial support: FIND (Geneva, Switzerland) made this supplement possible, with funding provided by the German Federal Ministry of Economic Cooperation and Development and the Canadian Department of Foreign Affairs, Trade, and Development.

Disclosure: The views expressed in this supplement are solely those of the author(s) and do not necessarily reflect the positions or policies of their affiliated institutions.

